# Comprehensive Immunohistochemical Study of the SWI/SNF Complex Expression Status in Gastric Cancer Reveals an Adverse Prognosis of SWI/SNF Deficiency in Genomically Stable Gastric Carcinomas

**DOI:** 10.3390/cancers13153894

**Published:** 2021-08-02

**Authors:** Marie-Isabelle Glückstein, Sebastian Dintner, Tim Tobias Arndt, Dmytro Vlasenko, Gerhard Schenkirsch, Abbas Agaimy, Gernot Müller, Bruno Märkl, Bianca Grosser

**Affiliations:** 1 Institute of General Pathology and Molecular Diagnostics, University Hospital Augsburg, 86156 Augsburg, Germany; Marie-Isabelle.Glueckstein@uk-augsburg.de (M.-I.G.); sebastian.dintner@uk-augsburg.de (S.D.); tobias.arndt@uka-science.de (T.T.A.); bruno.maerkl@uka-science.de (B.M.); 2 Institute of Mathematics and Computational Statistics, University of Augsburg, 86159 Augsburg, Germany; gernot.mueller@math.uni-augsburg.de; 3 Department of General, Visceral and Transplantation Surgery, University Hospital Augsburg, 86156 Augsburg, Germany; dmytro.vlasenko@uk-augsburg.de; 4 Tumor Data Management, University Hospital Augsburg, 86156 Augsburg, Germany; Gerhard.Schenkirsch@uk-augsburg.de; 5 Institute of Pathology, Friedrich-Alexander-University Erlangen-Nürnberg, University Hospital Erlangen, 91054 Erlangen, Germany; Abbas.Agaimy@uk-erlangen.de

**Keywords:** gastric cancer, SWI/SNF, immunohistochemistry

## Abstract

**Simple Summary:**

This study aimed to investigate the clinical relevance of immunohistochemical expression of proteins of the SWI/SNF complex, SMARCA2, SMARCA4 SMARCB1, ARID1A, ARID1B, and PBRM1 in 477 adenocarcinomas of the stomach and gastroesophageal junction. Additionally, the tumors were classified immunohistochemically in analogy to The Cancer Genome Atlas (TCGA) classification. Overall, 32% of cases demonstrated aberrant expression of the SWI/SNF complex. SWI/SNF aberration emerged as an independent negative prognostic factor for overall survival in all patients and in genomically stable patients in analogy to TCGA. In conclusion, determination of SWI/SNF status could be suggested in routine diagnostics in genomically stable tumors to identify patients who might benefit from new therapeutic options.

**Abstract:**

The SWI/SNF complex has important functions in the mobilization of nucleosomes and consequently influences gene expression. Numerous studies have demonstrated that mutations or deficiency of one or more subunits can have an oncogenic effect and influence the development, progression, and eventual therapy resistance of tumor diseases. Genes encoding subunits of the SWI/SNF complex are mutated in approximately 20% of all human tumors. This study aimed to investigate the frequency, association with clinicopathological characteristics, and prognosis of immunohistochemical expression of proteins of the SWI/SNF complexes, SMARCA2, SMARCA4 SMARCB1, ARID1A, ARID1B, and PBRM1 in 477 adenocarcinomas of the stomach and gastroesophageal junction. Additionally, the tumors were classified immunohistochemically in analogy to The Cancer Genome Atlas (TCGA) classification. Overall, 32% of cases demonstrated aberrant expression of the SWI/SNF complex. Complete loss of SMARCA4 was detected in three cases (0.6%) and was associated with adverse clinical characteristics. SWI/SNF aberration emerged as an independent negative prognostic factor for overall survival in genomically stable patients in analogy to TCGA. In conclusion, determination of SWI/SNF status could be suggested in routine diagnostics in genomically stable tumors to identify patients who might benefit from new therapeutic options.

## 1. Introduction

Gastric cancer is ranked as the sixth-most-common cancer entity worldwide, having accounted for approximately 780,000 cancer-associated deaths in 2018 [1]. So far, the best parameter for predicting prognosis and, therefore, therapy in gastric-cancer patients is TNM staging. The factors that are relevant for determining the prognosis of gastric carcinomas are local infiltration depth, locoregional lymph node involvement, distant metastases, and vascular invasion [2,3,4]. Additionally, diffuse Laurén subtype and proximal tumor localization are also known negative prognostic factors [5,6,7]. The introduction of perioperative chemotherapy after 2005 has improved the outcome in stage two and three gastric cancers, with a median survival of 50 months vs. 34 months [8]. However, the prognosis of gastric cancer is still poor and has a five-year-survival rate that has not changed during the period between 2000 and 2014, with survival rates being between 31.4% and 33.5% in Germany [9].

Two generally accepted molecular classifications have been proposed for gastric carcinomas, which have both prognostic and therapeutic implications, namely The Cancer Genome Atlas (TCGA) and the Asian Cancer Research Group (ACRG) classification [10,11]. So far, only a few prognostic and therapeutic biomarkers have been identified for gastric cancer. To date, the most important therapeutic marker in gastric carcinoma is HER2 overexpression [12]. In addition, MSI status and high PDL1 expression are independent positive prognostic factors in gastric carcinoma [13,14,15], while aberrant E-cadherin expression is considered an unfavorable prognostic factor and even a negative predictive factor for chemotherapy response [16].

SMARCA2, SMARCA4, SMARCB1, ARID1A, ARID1B, and PBRM1 are subunits of the Switch/Sucrose non-fermenting (SWI/SNF) complex, which show frequent alterations in rhabdoid tumors, ovarian clear cell carcinomas (OCCCs), and small-cell carcinoma of the ovary, hyper-calcemic type (SCCOHT) [17,18]. Numerous studies have demonstrated that this complex plays a role in tumor suppression in human cancers. Mutations or deficiencies of one or more subunits can have an oncogenic effect and influence the development, progression, and eventual therapy resistance of tumor diseases [18,19,20]. A few studies have also already demonstrated loss or heterogeneous expression patterns of these subunits in gastric carcinoma, which could make them potential starting points for new therapeutic concepts [21,22,23,24,25].

The aim of this retrospective study was to determine whether and to what extent molecular aberrations of SWI/SNF complex subunits play a role in gastric cancer in a large Western cohort. For this purpose, we evaluated the frequency, association with clinicopathological characteristics, and prognosis of alterations in SMARCA2, SMARCA4, SMARCB1, ARID1A, ARID1B, and PBRM1 in 477 carcinomas of the stomach and gastroesophageal junction. In addition, association with the subgroups of the molecular TCGA classification was investigated. Furthermore, determination of SWI/SNF status was reduced to SMARCA2, SMARCA4, SMARCB1, and ARID1A expression to facilitate applicability in routine diagnostics.

## 2. Materials and Methods

### 2.1. Patients

Surgical resection specimens from 511 patients with adenocarcinomas of the stomach and the gastroesophageal junction that were treated between 2005 and 2018 at the Department of Visceral Surgery of the University Hospital Augsburg were included in the study (AEGII and III according to Siewert and Stein [26]). Tumors from 34 patients were excluded from the study, because of low tumor percentage on the tissue microarray (TMA) and the final cohort consisted of 477 tumors. Of these, 347 tumors were treated with surgery alone, and 130 patients received neoadjuvant chemotherapy. Detailed clinical characteristics are summarized in Table 1.

Response to preoperative chemotherapy was determined histopathologically and was classified into three tumor regression grades (TRGs): TRG1b, TRG2, and TRG3, which corresponded to <10%, 10–50%, and >50% residual tumor cells [27]. Patients with TRG1b were classified as responders and with TRG2 and TRG3 as non-responders. Patients were treated with platinum/5-fluorouracil (5FU)-based chemotherapeutic regimes (Table 1). All surgical approaches included an abdominal D2-lymphadenectomy [28].

Follow-up data were obtained from the tumor data management of the University Hospital of Augsburg. Median follow-up was calculated by the inverse Kaplan–Meier method [29]. The primary endpoint of the study was overall survival (OS), which was defined as the time between the date of diagnosis and death by any cause.

The study was approved by the Institutional Review Board at the Ludwig-Maximilians-University of Munich (reference: 20-0922) and was performed in accordance with the Declaration of Helsinki.

### 2.2. Tissue Microarray Construction

All eligible histological sections were first re-evaluated using a light microscope (Olympus, Shinjuku, Japan) to verify the diagnosis. Representative slides of each tumor were digitalized using a Pannoramic SCAN II scanner (3DHISTECH, Budapest, Hungary), and five areas, consisting of normal tissue (1×), central tumor (2×), and tumor invasion front (2×), were selected. Based on the marked areas, formalin-fixed, paraffin-embedded (FFPE) tumor samples were subsequently automatically assembled into a tissue microarray (TMA) using a TMA Grandmaster (3DHISTECH, Budapest, Hungary) with a core size of 1 mm.

### 2.3. Immunohistochemistry and In Situ Hybridization

Immunohistochemical staining was performed on 2 µm sections from each TMA using primary antibodies listed in Supplementary Table S1. For PMS2, E-cadherin, CK7, CK20, CDX2, and EMA, a Ventana BenchMark ULTRA platform with an iVIEW DAB detection system was used (Roche, Mannheim, Germany). Staining for p53, SMARCA2, SMARCA4, SMARCB1, ARID1A, ARID1B, PBRM1, and MSH6 was performed on a BOND Rx platform with a BOND Polymer Refine Detection kit (Leica Biosystems, Nussloch, Germany). EBV-positive (EBV^+^) cases were identified by chromogenic in situ hybridization (EBER-CISH) likewise with the Ventana BenchMark ULTRA platform (Roche, Mannheim, Germany). Adequate controls were used for quality control of staining.

The stained sections were again digitalized, and the evaluation was performed with 3DHISTECH Casviewer (3DHISTECH, Budapest, Hungary) by one pathologist (Bianca Grosser) and one trained researcher (Marie-Isabelle Glückstein.). Discrepant cases were discussed with a senior pathologist (Bruno Märkl), and a consensus was established. The investigators were blinded both to the clinicopathological data and outcome.

Immunohistochemical expression of SMARCA2, SMARCA4, SMARCB1, ARID1A, ARID1B, and PBRM1 was classified as retained, reduced, loss, or hybrid-loss [21]. A strong homogeneous nuclear staining of non-neoplastic cells served as internal control. Reduced expression was defined as homogenous, very weak, but still recognizable, staining compared to normal cells. Tumors with hybrid loss showed loss of expression only in a subset of cells. Specimens lacking strong staining in the background of normal cells were not assessed [21,30].

### 2.4. TCGA Classification

Tumors were classified in analogy to TCGA-classification [10] as proposed by Setia et al. and Ahn et al. [31,32] in EBV^+^, mismatch repair deficient (MMRD), genomically stable (GS), and chromosomally instable (CIN) cases. Cases that showed nuclear staining by EBER-CISH were considered EBV^+^. The presence of MMRD was stated in case of loss of nuclear expression of MSH6 or PMS2. GS cases were identified according to aberrant E-cadherin expression. E-cadherin was considered positive if membranous staining was present in more than 50% of tumor cells [33]. Tumors were classified as CIN if an aberrant p53 expression pattern was present. p53 expression was considered aberrant if tumor cells showed complete loss of nuclear expression or if they showed staining with strong intensity in more than 60%. Staining of less than 60% with weak to moderate intensity was considered a wild-type expression pattern [34,35]. Cases that did not meet the above criteria were designated as unclassifiable.

### 2.5. Statistical Analysis

Chi-squared tests were used for hypothesis testing of differences between the relative frequencies. Kaplan–Meier estimates of survival rates were compared by log rank tests. Relative risks were estimated by hazard ratios (HR) from Cox proportional hazard models. Statistical analyses were performed using SPSS, Version 24 (IBM Corp., Armonk, NY, USA) and R Version 4.0.3. Exploratory 5% significance levels (two-tailed) were used for hypothesis testing.

## 3. Results

### 3.1. Cohort

The final cohort consisted of 477 patients with adenocarcinoma of the stomach or the gastroesophageal junction (Table 1). Of these, 130 patients received neoadjuvant chemotherapy and 347 underwent primary resection. The mean age of the cohort was 70.0 (range: 30.0–95.0) years, median follow up was 58.0 (range: 49.9–66.1) months; 52% of patients died during the follow-up period. Detailed clinicopathological characteristics are shown in Table 1.

### 3.2. SMARCA4 Expression

**Figure 1:**
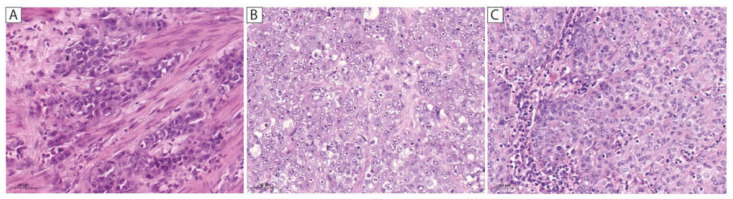
H&E (hematoxylin and eosin) images of the three cases with complete loss of SMARCA4 expression. All three cases (**A**–**C**) show an anaplastic solid and rhabdoid growth pattern. (**A**) The tumor shows overexpression of p53, expression of CK7, CK20, CDX2, EMA, and no expression of vimentin. (**B**) In this case expression of SMARCA2 and E-cadherin is completely lost. CK7, CK20, and CDX2 are not expressed whereas vimentin and EMA are expressed. (**C**) This tumor shows complete loss of E-Cadherin, expression of CK20, CDX2, EMA and no expression of CK7 and vimentin. Scale bar = 50 µm.

Analyses showed complete loss of SMARCA4 expression in three cases (0.6%). All tumors show an anaplastic solid and rhabdoid growth pattern (Figure 1), advanced T-stages, lymph node, and liver metastases. The median survival was eight (range: 7–12) months. Two patients received neoadjuvant chemotherapy and showed no response (TRG3). Two are subclassified as GS and one as CIN in analogy to the TCGA classification. All three cases were localized at the gastroesophageal junction (AEGII). Seven additional tumors (1.4%) showed reduced SMARCA4 expression. In all three cases with SMARCA4 loss, the expression of ARID1A and SMARCB1 was intact, whereas SMARCA2 was reduced in two of them and completely lost in one case as well. In one case, tumor cells turned out to be negative with both CK7 and CK20, whereas vimentin was expressed with a distinct perinuclear dot-like pattern. In addition, there was no expression of CDX2, and EMA was expressed in all three cases with SMARCA4 loss.

### 3.3. SMARCA2, SMARCB1, and ARID1A Expression

**Figure 2:**
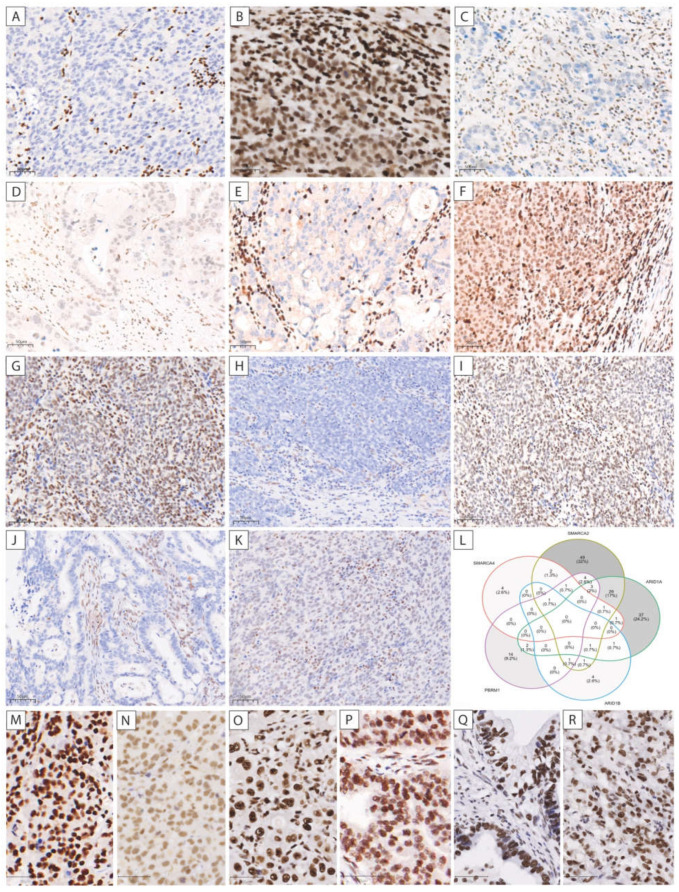
Representative images of immunohistochemical expression patterns of the proteins of the SWI/SNF complex. Complete loss of SMARCA4 (**A**), SMARCA2 (**C**), ARID1A (**E**), ARID1B (**H**), PBRM1 (**J**), and reduced expression of SMARCA4 (**B**), SMARCA2 (**D**), ARID1A (**F**), SMARCB1 (**G**), ARID1B (**I**), and PBRM1 (**K**) are shown. Venn-diagram of patients with loss or reduced expression of one of the proteins of the SWI/SNF complex (**L**). Retained expression patterns for SMARCA4 (**M**), SMARCA2 (**N**), ARID1A (**O**), SMARCB1 (**P**), ARID1B (**Q**), and PBRM1 (**R**) are presented. Scale bar = 50 μm.

Complete loss of ARID1A was observed in 59 (12.5%) and reduced expression in 11 (2.3%) cases. SMARCA2 expression was lost in 25 (5.4%) tumors and reduced in 65 (13.9%) cases. No case showed complete loss of SMARCB1. However, a heterogeneous expression pattern was detected in one case (0.2%). In addition, five cases (1.1%) showed reduced expression. Complete loss of PBRM1 occurred in 26 cases (5.5%) for PBRM1 and nine cases (1.9%) for ARID1B. Because both PBRM1 (31.4%), and ARID1B (49.8%) showed a very high proportion of cases with reduced expression, only cases with complete loss were designated as aberrant for consideration of the SWI/SNF status. Representative images of aberrant expression patterns are shown in Figure 2A–K and images of all cases with complete loss are presented in Supplementary Figure S1. In Figure 2M–R retained expression patterns of the proteins are shown.

As shown in Figure 2L, the largest overlap of aberrant expression can be seen between aberrant expression of SMARCA2 and ARID1A in 26 cases. One case showed simultaneous aberration of SMARCA4, SMARCA2, ARID1B, and PBRM1. No case with aberration of all the proteins involved in the SWI/SNF complex could be detected.

In cases of SMARCA2 loss, parallel loss of ARID1A occurred nine times. Seven of these nine cases showed intestinal type according to Laurén.

Regarding clinicopathological characteristics, complete loss of ARID1A was significantly associated with MMRD (*p* < 0.001) and aberrant expression with intestinal Laurén subtype (*p* = 0.014). Aberrant SMARCA2 expression was most frequently found in EBV^+^ tumors (*p* < 0.001), advanced tumor stages (*p* = 0.019), and patients with positive lymph nodes (*p* = 0.043). SMARCA2, (*p* = 0.160), ARID1A (*p* = 0.097), SMARCB1 (*p* = 0.930), PBRM1 (*p* = 0.520), and ARID1B (*p* = 0.490) expression showed no association with survival. Corresponding Kaplan–Meier curves are presented in Supplementary Figure S2.

### 3.4. SWI/SNF Status, Clinicopathologic Characteristics, and Survival

In the following, patients are classified according to the expression of the SWI/SNF proteins. If any of the proteins SMARCA2, SMARCA4, SMARCB1, or ARID1A showed reduced expression or loss, the case was designated as SWI/SNF-aberrant (SWISNFab). The cases with reduced and lost expression were combined because no survival difference was observed between the two groups (*p* = 0.452) (Supplementary Figure S3). Only complete loss of PBRM1 or ARID1B expression was considered aberrant.

Cases with SWISNFab were associated with advanced T-stage and MMRD subtype (each *p* < 0.01) and in CTx patients with low chemotherapy response (*p* = 0.023). Other associations with clinicopathological characteristics are presented in Table 1.

**Figure 3:**
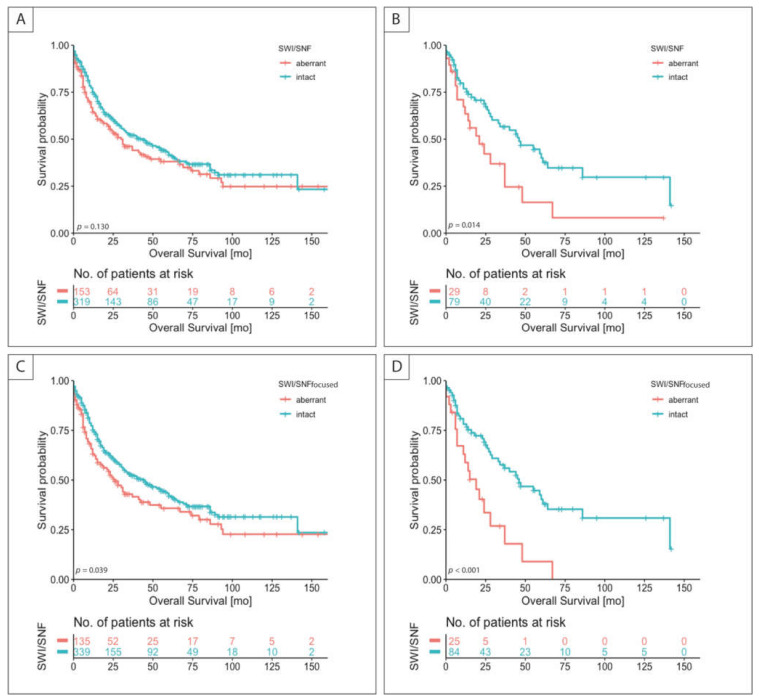
Discrimination of patient survival by SWI/SNF and SWI/SNFfocused status. Kaplan–Meier curves of patients with aberrant and intact proteins of the SWI/SNF complex are shown. SWI/SNF in all cases (*n* = 472) (**A**), in tumors classified as GS (*n* = 108) (**B**) and SWI/SNFfocused in all cases (*n* = 473) (**C**), and in GS tumors (*n* = 109) (**D**); *p*-value of log-rank test.

In the overall cohort no significant survival difference regarding the SWI/SNF status could be observed (*p* = 0.130) (Figure 3A). SWISNFab patients had a median survival of 30.0 (range: 19.3–40.8) months compared to cases with intact expression with 44.0 (range: 30.6–57.4) months.

In the subgroup analyses, the prognostic effect of SWISNFab was seen especially in tumors that were classified as GS in analogy to the TCGA classification (*p* < 0.014) (Figure 3B). SWISNFab patients had a median survival of 21.0 (range: 8.5–33.5) months compared to cases with intact expression with 46.0 (range: 23.8–68.2) months. In Cox regression analysis (Table 2), including known prognostic parameters, SWISNFab emerged as an independent prognostic factor for overall survival (HR 1.90, CI 1.04–3.50, *p* = 0.039) in GS tumors. In the other subgroups, no survival difference was seen with respect to SWI/SNF status. The corresponding survival curves are presented in Supplementary Figure S3. The SWISNF expression status and survival of subgroups according to TCGA can be found in Supplementary Figure S4.

### 3.5. Determination of SWI/SNF Status Using a Focused Panel of Protein Expression

To verify whether it is possible to reduce the panel for determining SWI/SNF status, only aberrant expression of SMARCA2, SMARCA4, SMARCB1, and ARID1A was considered and designated as SWI/SNFfocused.

Compared with SWI/SNF status, 18 patients were thus not classified as aberrant (Figure 2L). With regard to clinicopathologic characteristics, no essential difference was detected between SWI/SNF and SWI/SNFfocused (Supplementary Table S2).

As shown for SWI/SNFab, aberrant SWI/SNFfocused status emerged as an independent prognostic factor in GS tumors (HR 2.26, 95% CI 1.19–4.28, *p* = 0.012), and additionally also in the overall patient cohort (HR 1.42, 95% CI 1.03–1.95, *p* = 0.03) (Figure 3C,D, Supplementary Table S3).

## 4. Discussion

To the best of our knowledge, no study has evaluated the clinical relevance of the SWI/SNF complex in a large Western cohort of gastric cancer patients [21,22,23,24,36].

This study addressed this issue and analyzed the clinicopathological and prognostic relevance of the SWI/SNF complex in gastric adenocarcinomas with or without neoadjuvant CTx. We additionally investigated the association of the SWI/SNF complex with molecular subgroups in analogy to the TCGA.

The SWI/SNF complex has important functions in the mobilization of nucleosomes and consequently influences gene expression. Genes encoding subunits of the SWI/SNF complex are mutated in approximately 20% of all human tumors [18,19,20,37]. In our cohort, 32% of cases showed alteration of at least one of the subunits of the SWI/SNF complex, namely SMARCA2, SMARCA4, SMARCB1, ARID1A, PBRM1, and ARID1B.

Alterations of SMARCA4 occur in very low frequencies in solid tumors. We observed a loss of SMARCA4 expression in three cases (0.6%). Similar frequencies have been observed in other studies in gastric, esophageal, and lung carcinomas [22,24,30,38]. All three tumors with SMARCA4 deficiency showed very similar, specific histopathologic features and were located at the gastroesophageal junction (AEGII). Additionally, they showed very adverse clinical characteristics and poor survival. The specific growth pattern and clinical significance have been described by Agaimy et al. [21] in two cases and Huang et al. [22] in six cases. As described by Agaimy et al. [21] the expression pattern of cytokeratins was different among the SMARCA4-deficient cases. In the case of SMARCA4 loss, epithelial membrane antigen (EMA) seems to be an adequate marker to proof epithelial differentiation, whereas vimentin showed only in one case, a typical perinuclear dot-like pattern [39]. Furthermore, we observed >50% residual tumor (TRG3) in the two cases with SMARCA4 deficiency and preoperative CTx. As in adenocarcinomas of the lung and esophagus, no case with complete loss of SMARCB1 could be detected, suggesting that this subunit of the SWI/SNF complex does not play an overly important role in gastric carcinomas [24,30,38].

SWI/SNF alteration within at least one of its subunits was an independent negative prognostic factor for overall survival. This is totally in line with a very recently published large Asian gastric cancer cohort study, where SWI/SNF was altered in 35% of carcinomas and associated with a negative prognostic effect of altered SWI/SNF mainly in non-MSI/EBV diffuse type gastric carcinomas. Lacking data according to the molecular classification, this study could not further subclassify this non-MSI/EBV type [24]. Interestingly, we identified the GS group as mainly influenced by alterations of the SWI/SNF complex. The already-poor prognosis of this group that accounts for 23% of cases in our study was dramatically worsened in the SWSNFab group with a median survival time of 21 versus 46 months. In the GS subgroup 29 (19%) cases showed altered SWI/SNF. In contrast to the Asian study, we did not observe a survival difference in the subgroup analyses according to Laurén subtypes [24]. There might be an overlap with the diffuse type as 83% of the carcinomas in our GS subgroup were classified as diffuse type. Regarding the relatively high percentage of SWI/SNF-deficient carcinomas and the poor prognosis especially in the GS subtype, there is urgent need for new therapeutic strategies.

For *ARID1A*-deficient cancer cells, Ogiwara et al. [40] showed that they express low levels of gluthation (GSH), which makes them specifically vulnerable to inhibition of the GSH metabolic pathway. Additionally, increased sensitivity of *ARID1A*-deficient cancer cells to treatment with small molecule inhibitor of the PI3K/AKT pathway or selective sensitivity of EZH2 inhibitors against A*RID1A*-deficient gastric cancer could be demonstrated [41,42,43]. EZH2 inhibitor tazemostat is currently investigated in ongoing clinical trials including SMARCA4-negative solid tumors [18]. The most promising potential therapeutic option so far seems to be the sensitivity to double-strand DNA breaks inducing agents like PARP inhibitors because of the impairment of the DNA damage checkpoint [44]. PARP inhibitors are currently evaluated in several ongoing clinical trials. Furthermore, Shorstova et al. [45] found SWI/SNF compromised cancers to be susceptible to bromodomain inhibitors. An allosteric inhibitor of SMARCA2 and SMARCA4 has demonstrated anti-proliferative activity in a mouse xenograft model of *SMARCA4*-mutant lung cancer [20]. Initial studies showed the potential efficiency of checkpoint inhibitors and promotion of anti-tumor immunity in SWI/SNF-deficient tumors [46,47]. Interestingly, *SMARCB1*-mutant rhabdoid tumors and *SMARCA4*-mutant small cell carcinoma of the ovary have an immune active microenvironment and are responsive to immune-checkpoint inhibition [18,48,49]. We could also observe a strong association of SWI/SNF deficiency with MMRD subtype, for which checkpoint-inhibition is already a therapeutic option [50,51].

The analysis of a reduced panel of proteins to determine the SWI/SNF status proved to be almost equivalent in terms of determining the prognosis. Especially with regard to a possible determination of SWI/SNF status in routine diagnostics, it seems reasonable to limit the determination of SWI/SNF status in gastric cancer to SMARCA4, SMARCA2, and ARID1A.

Despite the comprehensive analysis of a large cohort, our study has limitations, which are mainly related to its retrospective nature. Our study has to be considered as an exploratory analysis and the results have to be validated in independent prospective cohorts. To elucidate the underlying molecular mechanisms of the alteration, we identified on the protein expression level, that sophisticated genetic and epigenetic investigations are necessary.

## 5. Conclusions

In summary, the expression of SMARCB1, does not appear to be of major importance in gastric carcinoma. The determination of SWI/SNF status with analyses of SMARCA2, SMARCA4, and ARID1A could be considered in routine practice, especially in the GS subgroup according to TCGA, to identify patients who might potentially benefit from new therapeutic alternatives.

## Figures and Tables

**Table 1:** Clinocopathological characteristics and SWI/SNF status.

Variable	*n* = 477 *		SWI/SNF-Aberrant (*n* = 153)		SWI/SNF Intact (*n* = 319)		*p* Value
Median age (range) (years)	70.0 (30.0–95.0)		72.0 (41.0–95.0)		68.0 (30.0–94.0)		0.193
Median survival (range) (months)	58.0 (49.9–66.1)		54.0 (34.7–73.3)		60.0 (53.5–66.5)		
Sex							0.121
Female	165	35%	61	40%	104	33%	
Male	312	65%	92	60%	215	67%	
T status							0.005
pT1/2	159	33%	38	25%	121	38%	
pT2/3	318	67%	115	75%	198	62%	
N status							0.448
Negative	178	37%	53	35%	122	38%	
Positive	299	63%	100	65%	197	62%	
Distant Metastasis							0.409
No	247	52%	75	49%	171	54%	
Yes	197	41%	66	43%	127	40%	
NA	33	7%	12	8%	21	7%	
Grading							0.471
Low grade	162	34%	48	31%	114	36%	
High grade	304	64%	100	65%	204	64%	
NA	11	2%	5	3%	1	0%	
Lymphovascular invasion							0.156
Negative	287	60%	85	56%	199	62%	
Positive	190	40%	68	44%	120	38%	
Vascular invasion							0.922
Negative	401	84%	128	84%	268	84%	
Positive	76	16%	25	16%	51	16%	
Laurén							0.159
Intestinal	266	56%	93	61%	172	54%	
Non-intestinal	211	44%	60	39%	147	46%	
Localization							0.471
Proximal	124	26%	36	24%	85	27%	
Non-proximal	335	70%	111	73%	222	70%	
NA	18	4%	6	4%	12	4%	
R status							0.508
R0	403	84%	131	86%	268	84%	
R1	54	11%	15	10%	38	12%	
Rx	20	4%	7	5%	13	4%	
TCGA							<0.001
EBV^+^	25	5%	14	9%	11	3%	
MSI	61	13%	40	26%	21	7%	
GS	110	23%	29	19%	79	25%	
CIN	151	32%	27	18%	123	39%	
No classification	130	27%	43	28%	85	27%	
Death							0.218
No	227	48%	67	44%	159	50%	
Yes	250	52%	86	56%	160	50%	
nCTx							0.972
No	347	73%	112	73%	234	73%	
Yes	130	27%	41	27%	85	27%	
TRG	*(n* = 130 **)		(*n* = 41)		(*n* = 85)		0.023
1b	10	7%	0	0%	9	11%	
2	37	28%	9	22%	28	33%	
3	83	63%	32	78%	48	56%	
CTx regimen							0.142
Cis/Ox + 5-FU or Cap	36	27%	14	34%	20	24%	
Ox + 5-FU + Doc	46	35%	11	27%	34	40%	
Cis + 5-FU + Epi	41	32%	12	29%	28	33%	
Ox + Epi + Cap	5	4%	2	5%	3	4%	
Others	2	2%	2	5%	0	0%	

*p*-values of Chi^2^-test are shown for difference between SWI/SNF aberrant and intact tumors. NA, not available; TCGA, The Cancer Genome Atlas; EBV^+^, EBV-positive; MSI, microsatellite instable; GS, genomically stable; CIN, chromosomally instable; nCTx, neoadjuvant chemotherapy; TRG, tumor regression grades; Cis, cisplatin; Ox, oxaliplatin; 5-FU, 5-fluorouracil; Cap, capecitabine; Doc, docetaxel; Pac, paclitaxel; Epi, epirubicin; Others, combination of Cis/Ox with other agents or cross over between different treatment regimens; * five patients without information for SMARCA2, SMARCA4, SMARCB1, ARID1A, ARID1B, or PBRM1; ** four patients without information for SMARCA2, SMARCA4, SMARCB1, ARID1A, ARID1B, or PBRM1; italics values: Number of patients who received nCTx.

**Table 2:** Cox regression analyses of SWI/SNF status in all cases and in GS tumors.

Variable	Overall Survival (*n* = 472)				Overall Survival (*n* = 108)			
	HR	95% CI		*p*	HR	95% CI		*p*
T-status	1.829	1.266	2.643	0.001	1.898	0.734	4.911	0.186
N-status	1.708	1.209	2.414	0.002	2.664	1.212	5.853	0.015
Age	1.015	1.003	1.027	0.017	1.027	1.002	1.052	0.033
M-status	2.517	1.820	3.480	<0.001	2.045	1.027	4.073	0.042
R-status	1.977	1.368	2.857	<0.001	1.626	0.842	3.142	0.148
SWI/SNF	1.218	0.898	1.651	0.205	1.904	1.035	3.503	0.039
TCGA	-	-	-	0.440	-	-	-	-
EBV^+^	1.737	0.747	4.036	0.200	-	-	-	-
MSI	1.753	0.781	3.934	0.173	-	-	-	-
GS	2.047	0.921	4.552	0.079	-	-	-	-
CIN	1.670	0.744	3.751	0.214	-	-	-	-

CI, Confidence interval (95%); TCGA, The Cancer Genome Atlas; EBV^+^, EBV-positive; MMRD, mismatch repair deficiency; GS, genomically stable; CIN, chromosomal instable.

## Data Availability

The datasets generated during the current work are available from the corresponding author on reasonable request.

## Supplementary Materials

The following are available online at https://www.mdpi.com/article/10.3390/cancers13153894/s1, Figure S1: SWI/SNF expression status and survival in subgroups according to TCGA; Figure S2: Kaplan–Meier curves of the patients with aberrant, reduced, and intact expression of ARID1A (A), SMARCA2 (B), SMARCA4 (C), SMARCB1 (D), PBRM1 (E), and ARID1B (F) are shown; Figure S3: Survival in cases with complete and reduced expression of SWI/SNFfocused status; Figure S4: SWI/SNF expression status and survival in subgroups according to TCGA; Table S1: Antibodies and dilutions; Table S2: Clinicopathological characteristics and SWI/SNFfocused status; Table S3: Cox regression analysis of SWI/SNFfocused status.
