# Supplementary material for: Comprehensive Immunohistochemical Study of the SWI/SNF Complex Expression Status in Gastric Cancer Reveals an Adverse Prognosis of SWI/SNF Deficiency in Genomically Stable Gastric Carcinomas
Source: Cancers (Basel). 2021 Aug 2;13(15):3894. doi: 10.3390/cancers13153894 (PMC8345509; doi:10.3390/cancers13153894)
Supplement: Supplementary file 1 [file cancers-13-03894-s001.zip › cancers-1320894-supplementary.pdf]

cancers

MDPI

Supplementary Materials

Comprehensive Immunohistochemical Study of the SWI/SNF Complex Expression Status in Gastric Cancer Reveals an Adverse Prognosis of SWI/SNF Deficiency in Genomically Stable Gastric Carcinomas

Marie-Isabelle Glückstein, Sebastian Dintner, Tim Tobias Arndt, Dmytro Vlasenko, Gerhard Schenkirsch, Abbas Agaimy, Gernot Müller, Bruno Märkl and Bianca Grosser

Table S1. Antibodies and dilutions.

Antigen	Clone	Company	Dilution
ARID1A	EPR13501	Abcam (Cambridge, United Kingdom)	1:50
ARID1B	E1U7D	Cell Signaling Technology (Danvers, USA)	1:100
CDX2	EPR2764Y	Cell marque (Rocklin, USA)	RTU
CK7	SP52	Roche Diagnostics (Mannheim, Germany)	RTU
CK20	KS20.8	Cell marque (Rocklin, USA)	1:300
E-Cadherin	EP700Y	Cell marque (Rocklin, USA)	1:100
EBER	MRQ47	Cell marque (Rocklin, USA)	RTU
EMA	E291	Cell marque (Rocklin, USA)	1:200
MSH6	EP49	Leica Biosystems (Newcastle, UK)	RTU
PMS2	EP51	Agilent Technologies (Santa Clara, USA)	RTU
PBRM1	D4L9X	Cell Signaling Technology (Danvers, USA)	1:00
p53	DO-7	Roche Diagnostics (Mannheim, Germany)	RTU
SMARCA2	polyclonal	Sigma Aldrich (St. Louis, USA)	1:50
SMARCA4	Epncir111A	Abcam (Cambridge, United Kingdom)	1:100
SMARCB1	MRQ-27	Cell marque (Rocklin, USA)	1:50

Table S2. Clinicopathological characteristics and SWI/SNFfocused status.

Variable		*n* = 477 *		SWI/SNFfocused aberrant (*n* =135)		SWI/SNFfocused Intact (*n* = 339)		*p*-Value
Median age (range) [years]		70.0 (30–95)		72.0 (43.0–95.0)		68.0 (30.0–94.0)		0.186
Median survival (range) [months]		58.0 (49.9–66.1)		54.0 (37.1–76.9)		60.0 (53.5–66.5)		
Sex								0.087
	male	312	65%	80	59%	229	68%	
	female	165	35%	55	41%	110	32%	
T status								0.008
	pT1/2	159	33%	33	24%	126	37%	
	pT2/3	318	67%	102	76%	213	63%	
N status								0.510
	negative	178	37%	47	35%	129	38%	
	positive	299	63%	88	65%	210	62%	
Distant Metastasis								0.628
	no	247	52%	68	50%	178	53%	
	yes	197	41%	58	43%	137	40%	
	NA	33	7%	9	7%	24	7%	
Grading								0.316
	low grade	162	34%	39	29%	123	36%	
	high grade	304	64%	92	68%	209	62%	
	NA	11	2%	4	3%	7	2%	
Lymphovascular invasion								0.089
	negative	287	60%	73	54%	212	63%	
	positive	190	40%	62	46%	127	37%	
Vascular invasion								0.514

	negative	401	84%	111	82%	287	85%	
	positive	76	16%	24	18%	52	15%	
Lauren								0.282
	intestinal	266	56%	81	60%	185	55%	
	non-intestinal	211	44%	54	40%	154	45%	
Localization								0.676
	proximal	124	26%	33	24%	89	26%	
	non-proximal	335	70%	97	72%	237	70%	
	NA	18	4%	5	4%	13	4%	
R status								0.299
	R0	403	84%	116	86%	284	84%	
	R1	54	11%	12	9%	42	12%	
	Rx	20	4%	7	5%	13	4%	
TCGA								<0.001
	EBV+	25	5%	14	10%	11	3%	
	MSI	61	13%	38	28%	23	7%	
	GS	110	23%	25	19%	84	25%	
	CIN	151	32%	23	17%	127	37%	
	no classification	130	27%	35	26%	94	28%	
Death								0.133
	no	227	48%	57	42%	169	50%	
	death	250	52%	78	58%	170	50%	
Preoperative CTx								0.849
	no	347	73%	98	73%	249	73%	
	yes	130	27%	37	27%	90	27%	
TRG		*(n = 130 *)*		*(n = 37)*		*(n = 90)*		0.023
	1b	10	7%	0	0%	9	10%	
	2	37	28%	7	19%	29	32%	
	3	83	65%	30	81%	52	58%	
CTx regimen								0.140
	Cis/Ox + 5-FU or Cap	36	27%	12	32%	22	24%	
	Ox + 5-FU + Doc	46	35%	10	27%	35	39%	
	Cis + 5-FU + Epi	41	32%	11	30%	30	33%	
	Ox + Epi + Cap	5	4%	2	5%	3	3%	
	Others	2	2%	2	5%	0	0%	

p-values of Chi^2-test are shown for difference between SWI/SNF aberrant and intact tumors; TCGA: The Cancer Genome Atlas, EBV$^+$: EBV positive, MSI: microsatellite instable; GS: genomically stable; CIN: chromosomally instable; Cis, cisplatin; Ox, oxaliplatin; 5-FU, 5-fluorouracil; Cap, capecitabine; Doc, docetaxel; Pac, paclitaxel; Epi, epirubicin; Others, combination of Cis/Ox with other agents or cross over between different treatment regimens; *3 patients without information for SMARCA2, SMARCA4, SMARCB1, and ARID1A; italics values: Number of patients who received nCTx.

Table S3. Cox regression analysis of SWI/SNFfocused status.

	**Overall Survival (*n* = 474)**				**Overall survival (*n* =109)**			
	**HR**	**CI**		***p***	**HR**	**CI**		***p***
T-status	1.715	1.191	2.471	0.004	1.841	0.723	4.692	0.201
N-status	1.772	1.259	2.495	0.001	2.132	1.015	4.480	0.046
Age	1.045	1.031	1.059	<0.001	1.210	0.469	3.122	0.693
M-status	2.841	2.052	3.935	<0.001	1.994	0.991	4.012	0.053
R-status	2.399	1.659	3.469	<0.001	2.176	1.147	4.125	0.017
SWI/SNF	1.420	1.033	1.953	0.031	2.259	1.192	4.279	0.012
TCGA	-	-	-	0.173	-	-	-	-
EBV+	1.293	0.551	3.034	0.555	-	-	-	-
MSI	1.791	0.788	4.073	0.164	-	-	-	-
GS	2172	0.957	4.933	0.064	-	-	-	-
CIN	1819	0.794	4.171	0.157	-	-	-	-

CI: Confidence intervall (95%), TCGA: The Cancer Genome Atlas, EBV+: EBV positive, MSI: Microsatellite instability, GS: genomically stable, CIN: chromosomally instable.

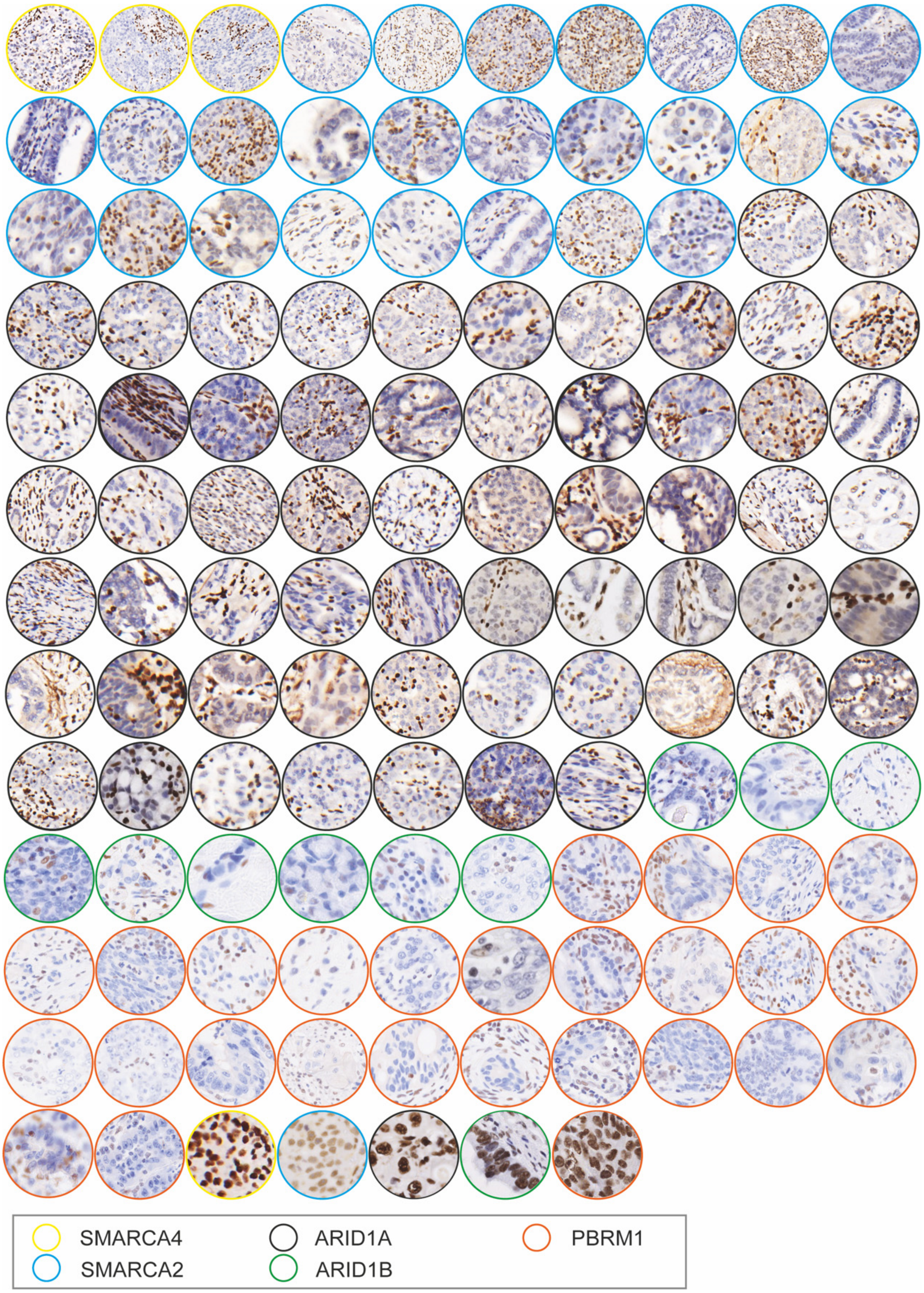

Figure S1. Images for all cases with complete loss of one of the proteins of the SWI/SNF complex at ×20 magnification. All cases with complete loss of SMARCA4, SMARCA2, ARID1A, ARID1B and PBRM1 are shown. The last five cores at the bottom show representative images with retained staining.

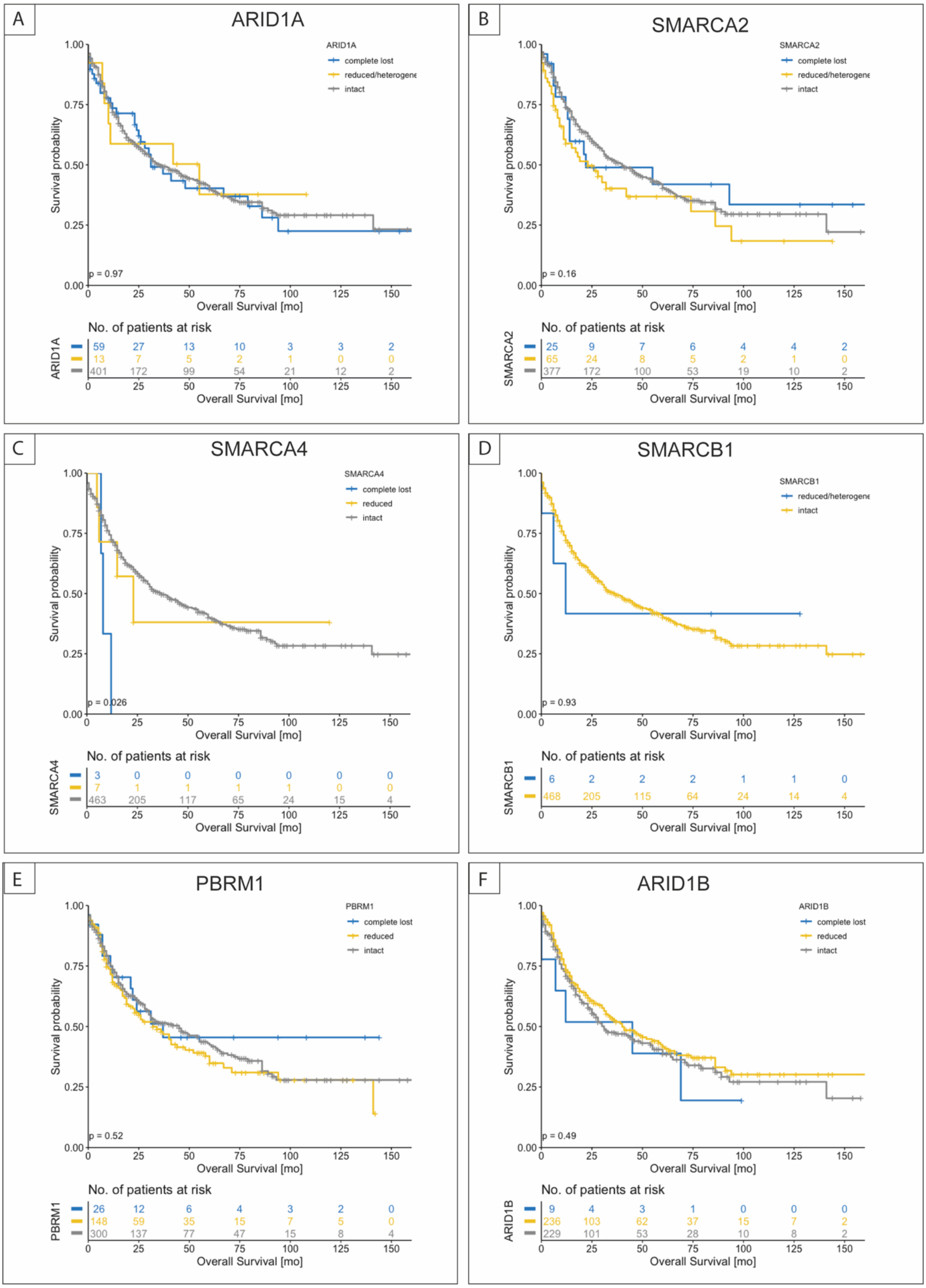

Figure S2. Kaplan-Meier curves of the patients with aberrant, reduced and intact expression of ARID1A (**A**), SMARCA2 (**B**), SMARCA4 (**C**), SMARCB1 (**D**), PBRM1 (**E**) and ARID1B (**F**) are shown. No., number; EBV^{+}, EBV positive; MMRD, mismatch-repair deficient; CIN, chrosomal instable; *p*-value of log-rank test (overall).

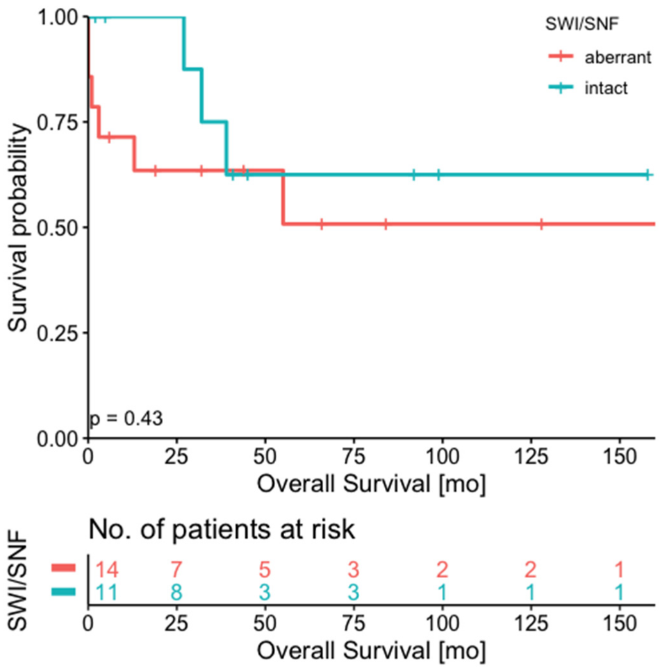

Figure S3. Survival in cases with complete and reduced expression of SWI/SNFfocused status. Kaplan-Meier curves of the patients with complete loss and reduced expression of SWI/SNFfocused complex are shown. No., number; *p*-value of log-rank test (overall).

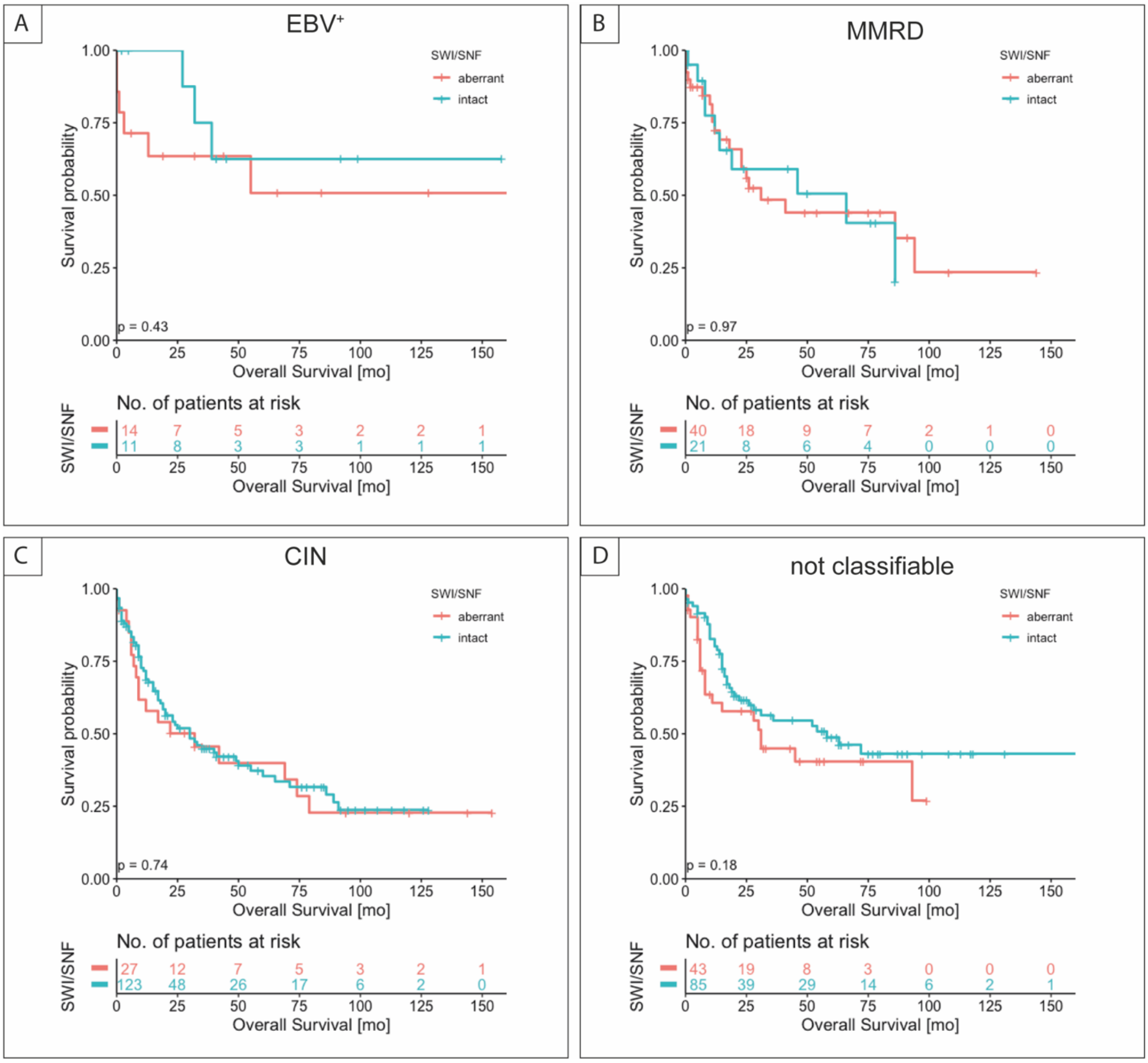

Figure S4. SWI/SNF expression status and survival in subgroups according to TCGA. Kaplan-Meier curves of the patients with aberrant and intact SWI/SNF complex in EBV+ (A), MMRD (B), CIN (C) and non-classifiable tumors (D) according to TCGA are shown. No., number; EBV+, EBV positive; MMRD, mismatch-repair deficient; CIN, chrosomal instable; *p*-value of log-rank test (overall).
